# Emergency Peripartum Hysterectomies at a District General Hospital in United Kingdom: 10-Year Review of Practice

**DOI:** 10.1155/2016/9875343

**Published:** 2016-04-12

**Authors:** J. Chester, P. Sidhu, S. Sharma, F. Israfil-Bayli

**Affiliations:** ^1^Sandwell and West Birmingham NHS Trust, City Hospital, Dudley Road, Birmingham B18 7QH, UK; ^2^Walsall Healthcare NHS Trust, Moat Road, Walsall WS2 9PS, UK

## Abstract

Peripartum haemorrhage is an obstetric emergency which requires effective and timely management. A retrospective analysis was conducted at a single centre district hospital, over a 10-year period to describe factors that would lead to a peripartum hysterectomy. We sought to establish intraoperative and postoperative risks and review outcomes and complications associated with the procedure. A total of 29 cases (incidence 0.8 per 1000) were reviewed over 2001–2011. The mean parity was 1.8 and the mean maternal age was 33 years. Uterine atony was the most common indication for hysterectomy (12/29) followed by placenta praevia and accreta (4/29 and 5/29 cases, resp.). The commonest postoperative complications were sepsis and paralytic ileus. EPH most commonly occurs due to uterine atony but remains difficult to predict. Hospitals should continue to have robust systems and the necessary resources available to perform EPH where clinically indicated.

## 1. Introduction

The maternal mortality rate in the United Kingdom (UK) is 11.39 deaths per 100,000 live births per year (95% CI 10.09–12.86) [1]. The 2006–2008 Confidential Enquiry into Maternal Deaths (CEMD) reported haemorrhage as the third most common cause of maternal death directly related to pregnancy, after thromboembolism and eclampsia [1]. The incidence of major obstetric haemorrhage in the UK is 3.7 per 1000 births (95% CI 3.4–4.0) [1]. Risk factors for postpartum haemorrhage are well known, including previous caesarean section, induction of labour, and retained placenta [2]. When severe bleeding occurs after delivery, whether vaginal delivery or caesarean section, multiple medical and surgical options are available. These include syntometrine, oxytocin, Bakri balloons, or uterine artery ligation [2]. Where these fail and bleeding becomes life threatening, then an emergency peripartum hysterectomy (EPH) is required. Fortunately, emergency peripartum hysterectomy is seldom performed. The serious nature of the procedure and its impact upon patients both physically and psychologically mean that it should remain a last resort after other methods have been exhausted. Awareness of risk factors, complications, and outcomes will be beneficial for ensuring that the possibility of major obstetric haemorrhage is considered during labour and appropriate steps are taken to minimise the risk.

This review analysed all emergency peripartum hysterectomies undertaken in a district general hospital in the UK over the last 10 years. The objective was to review emergency peripartum hysterectomies, establish intraoperative and postoperative risks, and review outcomes in our unit. We also compared our results with recent published literature to identify any current trends in the management of EPH.

## 2. Material and Methods

A retrospective review of the delivery suite theatre log identified all women undergoing EPH between Jan 2001 and Dec 2010. Trust approval was obtained to allow notes to be accessed and appropriate results and imaging reviewed. Clinical parameters such as age, parity, gestational age at delivery, method of delivery, indication for caesarean section, type of hysterectomy and perioperative complications, histological outcome, and postoperative short- and long-term follow-up were obtained from the patient notes and results systems and recorded using a standardised data form approved by the trust. Data were analysed and the results presented.

## 3. Results

Over the ten-year period reviewed 29 peripartum hysterectomies were performed, giving an incidence of 0.8 per 1000 deliveries. The modal parity was 1, with two women (6.8%) being grand multiparous (given birth 5 times or more) whilst two women (6.8%) were primigravida. The mean maternal age at delivery was 33.2 years and the mean gestational age at delivery was 37 + 6 weeks. There were 15 (51.7%) cases that required EPH following delivery by caesarean section, of which six were elective caesarean sections and nine emergency caesarean sections (see Table 1). The number of patients that required EPH after vaginal delivery was 14. Interestingly, over half the women had had one or more previous caesarean sections (*n* = 15, 51.7%) with six of these women (20.6%) having had two or more caesarean sections in the past. Further analysis of the patients who are in labour, irrespective of mode of delivery, demonstrated that seven cases (30.4%) were induced.

The most common indication for hysterectomy was uterine atony (*n* = 12, 41.3%). Placenta accreta and praevia were subsequently found in four (13.8%) and five (17.2) cases, respectively. The numbers of cases that received total and subtotal hysterectomy were 15 and 14, respectively (Table 1). There was no link between initial mode of delivery and type of hysterectomy performed, with the main factor influencing choice being ease of accessibility to the cervix.

Unsurprisingly bleeding was the commonest intraoperative complication occurring in 24 patients (82.8%). The only other complication experienced was injury to the ureter, occurring in four cases (13.8%). These were all identified and repaired intraoperatively. There were no cases of bowel or vascular injuries in the cases reviewed (Table 2).

As expected a high percentage of patients (82.8%, *n* = 24) required a blood transfusion intraoperatively. The mean quantity of blood transfused was 5.6 U of packed red cells.

The majority of patients required admission to the Obstetric High Dependency Unit postoperatively (*n* = 23, 79.3%). Over the ten years reviewed there were no events of maternal mortality. Postoperative complications occurred in ten cases (34.5%) with analysis demonstrating that paralytic ileus (*n* = 3, 10.3%) and sepsis (*n* = 2, 6.9%) were the commonest.

In total 86.2% (*n* = 25) women were followed up in clinic at 6 weeks by their consultant obstetrician. During this visit the patient was reviewed and debriefed regarding the intrapartum events. All patients underwent speculum examination to review the amount of cervical tissue remaining, to ensure correct information regarding the need for future cervical smears. Histology was reviewed and the patient referred for further support as necessary.

## 4. Discussion

Peripartum haemorrhage is a global source of maternal mortality [2]. Its incidence is significantly lower in developed countries, such as the UK, where a multidisciplinary approach alongside a supportive infrastructure in the hospital has been integral to reducing maternal deaths. Despite this, patients still die from bleeding in the postpartum period.

We aimed to review patients who underwent an EPH at a single centre to analyse potential causative factors associated with EPH, determine associated complications, and review outcomes.

The most common indication in the group studied for EPH was uterine atony. These represent cases which are hard to predict. Risk factors for uterine atony such as prolonged labour with or without a long duration of oxytocin augmentation should be identified during labour. Within our cohort 30% of those who are in labour were induced using standard prostaglandin analogues. As suggested in previous literature their use does seem to impact upon the risk of postpartum haemorrhage [2–5]. It is suggested that their use desensitises receptors which then respond less well to oxytocin postpartum leading to uterine atony [2–5]. The literature also suggests that EPH may be 2–4 times more common following caesarean section than vaginal delivery [2–5]. Our incidence of EPH in women who are in labour versus caesarean sections showed little difference; however without the use of intravenous oxytonics in caesarean section as recommended by NICE the rate may have been higher [6].

All these factors highlight the difficulty in identifying those who will require EPH. Although risk factors for PPH are well known and documented, knowing which of those will progress and required EPH is very difficult.

In all cases, use of pharmacological management was exhausted but use of surgical methods of uterine compression was not widely practiced. The Royal College of Obstetrics and Gynaecology green top guideline on PPH recommends the use of lesser surgical methods, as previously discussed, before resorting to EPH [2]. This highlights the need to ensure appropriate surgical training for all obstetricians in these methods to ensure they are utilised where possible. This will inevitably minimise the need for EPH and reduce morbidity.

Increasingly services such as interventional radiology are being used to provide a further therapeutic option before EPH. Embolisation of the uterine artery (UAE) has been shown to be effective, with a success rate of over 70% [7, 8]. Studies have found that this success rate is highest amongst vaginal deliveries, in cases of uterine atony and where patients are not shocked [9]. Pelage et al. showed less than 1% of patients undergoing UAE in cases of major haemorrhage proceeded to hysterectomy [8]. In larger centres interventional radiology services are often available out of hours; unfortunately this is not the case in our unit. Here UAE could be considered as an option in those planned cases such as placenta accreta providing a further management option before proceeding to EPH.

Within our hospital there were no guidelines as to when subtotal or total hysterectomy should be used, with choice being based solely on operator's choice and ease of access to the cervix. El-Jallad et al. suggest that total hysterectomy is beneficial, reducing the risk of future cervical cancer [10]. Supporters of subtotal hysterectomy state that it is associated with reduced blood loss, a shorter duration of operation, and fewer urinary tract injuries; however the evidence reviewed suggests this is not the case [11]. Our study found no significant difference between intraoperative complications, blood loss, and postoperative complications between the two methods. The RCOG does not explicitly recommend one specific method over the other [2].

Within our study the most common postoperative complications were sepsis and ileus. However these occurred in around 34% of cases. The VALUE study found complications occurring in only 8.3% of patients undergoing abdominal hysterectomy [12]. Our rate is significantly higher than would be expected and may be due to the emergency nature of the procedure. Both the unplanned aspect of EPH and its inherent need for quick action may result in higher levels of complications than would be experienced in elective surgery. The increased complication rate is important to remember both when discussing the operation with patients and relatives (where appropriate) and when considering using EPH. However, as discussed by the RCOG, recourse to EPH should not be left until the patient is perimoribund [2].

EPH is a very traumatic event with the potential for long-term physical and psychological sequellae. For most women the unplanned loss of the opportunity to extend their family has a major impact upon mental health. Following up patients to ensure thorough debriefing and discussion of events is essential. This provides a chance to ensure appropriate follow-up.

Although a last resort and rare, EPH is a lifesaving procedure and all obstetricians should obtain sufficient technical experience to perform this procedure when required. Most units recommend EPH to be decided upon and performed by two consultants due to the increased risks associated with this procedure both physically and psychologically. Most units require one of these to be an experienced gynaecologist with greater experience of complex hysterectomies. However this makes training opportunities for trainees limited.

Our data highlights that although uncommon, EPH is a procedure that can be complex but potentially lifesaving. Although a few cases had a clear prepartum risk factor for EPH (placenta accreta), most had no clear risk factor. This study shows the importance of obstetricians being alert to the possibility of EPH in all women. The need for appropriate resources and protocols to be in place for when EPH is required is essential in all obstetric units. EPH should be considered as a last resort when all management options have been exhausted.

## Figures and Tables

**Table 1:** Delivery details and type of hysterectomy.

	Incidence (*n*=)	(%)
Onset of labour		
Spontaneous	16	(55.1)
Induced	7	(24.1)
Elective caesarean section	6	(20.8)
Delivery method		
Vaginal delivery	14	(48.3)
Caesarean section	15	(51.7)
Elective	6	(20.8)
Emergency	9	(29.9)
Type of hysterectomy		
Subtotal	14	(48.3)
Total	15	(51.7)

**Table 2:** Intraoperative and postoperative complications.

Complication	Incidence (*n*=)	(%)
Intraoperatively		
Ureteric injury	4	(13.8)
Bleeding requiring transfusion	24	(82.8)
*Total*	*28*	*(96.6)*
Postoperative complications		
Sepsis	2	(6.9)
Acute tubular necrosis	1	(3.4)
Wound breakdown/infection	1	(3.4)
Ileus	3	(10.3)
Vault haematoma	1	(3.4)
Pelvic collection	1	(3.4)
Postop bladder problems	1	(3.4)
*Total*	*10*	*(34.5)*
